# A novel deep learning based hippocampus subfield segmentation method

**DOI:** 10.1038/s41598-022-05287-8

**Published:** 2022-01-25

**Authors:** José V. Manjón, José E. Romero, Pierrick Coupe

**Affiliations:** 1 Instituto de Aplicaciones de las Tecnologías de la Información y de las Comunicaciones Avanzadas (ITACA), Universitat Politècnica de València, Camino de Vera s/n, 46022 Valencia, Spain; 2 Univ. Bordeaux, LaBRI, UMR 5800, PICTURA, 33400 Talence, France; 3 CNRS, LaBRI, UMR 5800, PICTURA, 33400 Talence, France

**Keywords:** Biomarkers, Neurology, Mathematics and computing

## Abstract

The automatic assessment of hippocampus volume is an important tool in the study of several neurodegenerative diseases such as Alzheimer's disease. Specifically, the measurement of hippocampus subfields properties is of great interest since it can show earlier pathological changes in the brain. However, segmentation of these subfields is very difficult due to their complex structure and for the need of high-resolution magnetic resonance images manually labeled. In this work, we present a novel pipeline for automatic hippocampus subfield segmentation based on a deeply supervised convolutional neural network. Results of the proposed method are shown for two available hippocampus subfield delineation protocols. The method has been compared to other state-of-the-art methods showing improved results in terms of accuracy and execution time.

## Introduction

The hippocampus (HC) is a bilateral brain structure located in the medial temporal lobe at both sides of the brainstem near to the cerebellum. HC is involved in many brain functions such as memory and spatial reasoning^1^. It plays also an important role in many neurodegenerative diseases such as Alzheimer's disease (AD)^2^. Furthermore, hippocampus volume estimation is considered a valuable tool for follow-up and treatment adjustment^3–5^.

In the last years, many HC segmentation methods have been proposed^6–8^. Most of them, were restricted to consider the hippocampus as a single structure^9^ due image resolution limitations. However, it is well known that, for example, AD affects the different HC subfields at different moments during the disease progression^2,10^. Thus, automatic and accurate HC subfield segmentation methods would be really important to obtain early biomarkers of the disease.

Currently, advances in modern MR sequences allow acquiring high-resolution images making possible to divide the hippocampus into its constituent parts. In the last years, several delineation protocols have been proposed (some of these protocols have been used to create manually labeled MRI datasets). However, there is still little consensus between the different HC subfield protocols as shown in Ref.^11^ where 21 delineation protocols were compared. For example, in 2013, Winterburn presented a new in-vivo high-resolution atlas^12^ to divide the hippocampus in five different sub-regions: CA1, CA2-3, CA4/DG, Stratum and Subiculum. Later, in 2015, Kulaga-Yoskovitz developed another segmentation protocol^13^ consisting of three structures: CA1-3, CA4/DG and Subiculum.

Several automatic methods for HC subfield segmentation have been developed in the last years^14–16^. One of the most well-known methods for HC subfield segmentation is named ASHS^17^ that uses a multi-atlas approach combined with a similarity-weighted voting and a boosting-based error correction. Unfortunately, this method took several hours to produce a segmentation due to the exhaustive use of non-linear registrations (an updated version of this software has greatly reduced this time to few minutes). More recently, we proposed a method named HIPS^18^ that obtained state-of-the-art results in two different delineation protocols (Winterburn and Kulaga-Yoskovitz) with relatively low processing times thanks to the use of a fast multi-atlas label fusion method called OPAL^19^. Although these methods have promising results, their automatic measurements are not close enough to manual tracings in some cases^20^.

Recently, due to the expansion of deep learning in medical imaging, novel methods based on this technology have been proposed to further improve the accuracy of HC segmentation. For full hippocampus segmentation many methods based on convolutional neural networks (CNN) have been already proposed^21–24^. Recently, deep learning-based methods has been also proposed for hippocampus subfield segmentation. For example, UGNET has been proposed^25^ using an adversarial training approach and also variants of the famous UNET architecture^26^ such as the Dilated Dense UNET^27^ have been proposed. However, one of the major problems of supervised deep learning methods is their hunger for training data to be able to generalize on unseen data.

In this paper, we propose a novel deep-learning based segmentation method that takes benefit of a problem specific preprocessing that locates the data in a canonical geometrical and intensity space therefore simplifying the segmentation problem and thus reducing the need for lots of manually labeled data. The proposed method has been validated using two hippocampus subfield segmentation protocols with publically available datasets.

## Materials and methods

### Training data

**Figure 1:**
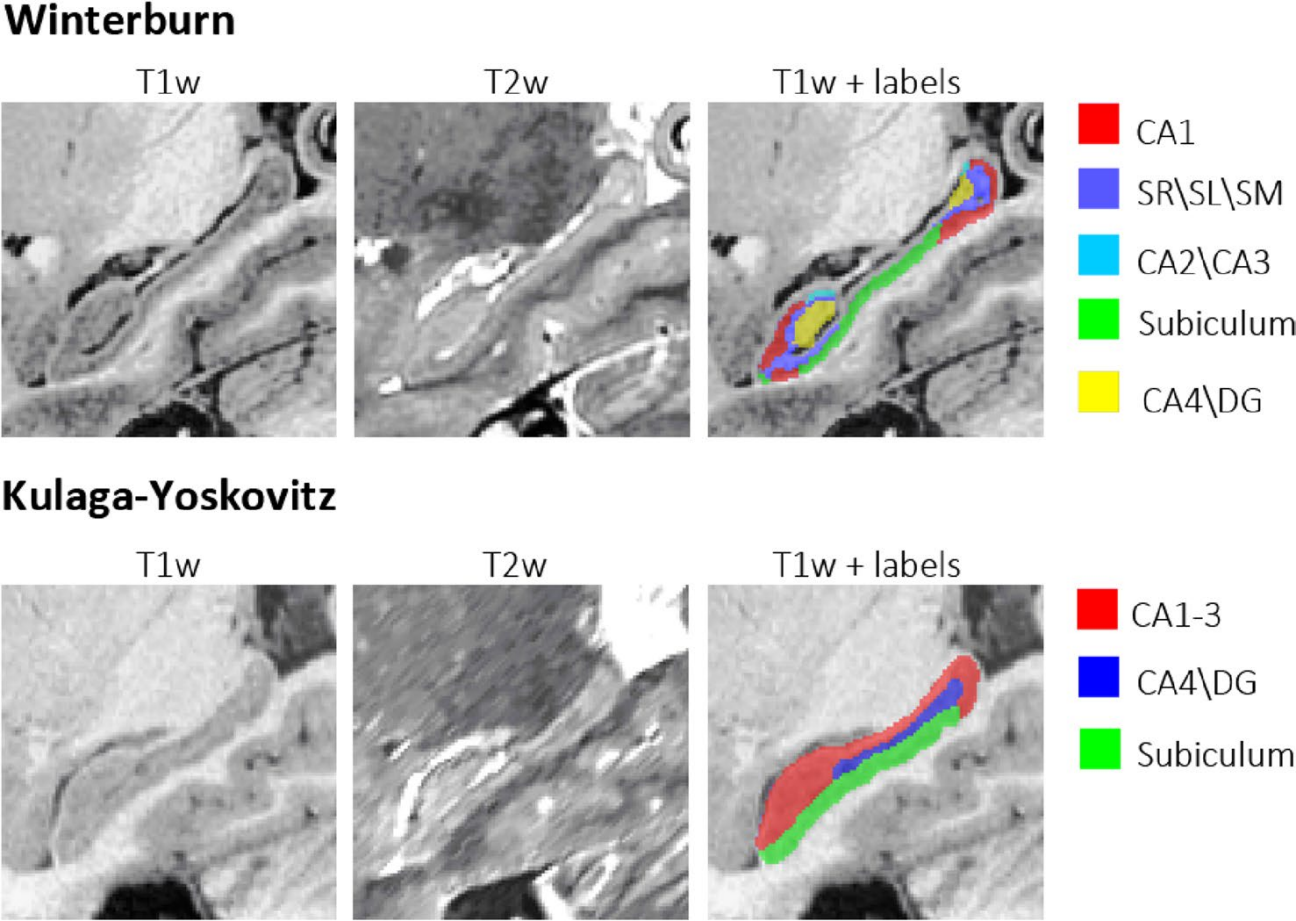
Examples from Winterburn and Kulaga-Yoskovitz datasets showing T1w, T2w and manual segmentations. Images generated using ITK-SNAP v 3.4.0.

In this work, we have used two different datasets including two manual labeling hippocampus subfield segmentation protocols, both with high-resolution (HR) T1w and T2w MR images (see Fig. 1). Details of these datasets are given below:

#### Kulaga-Yoskovitz dataset

This dataset includes 25 subjects from a public repository (http://www.nitrc.org/projects/mni-hisub25) (31 ± 7 years, 12 males, 13 females) with manually segmented labels dividing the HC in three parts (CA1-3, DG-CA4 and Subiculum). The Ethics Committee of the Montreal Neurological Institute and Hospital approved the study and written informed consent was obtained from all participants in accordance with the standards of the Declaration of Helsinki. Participants gave their written informed consent prior to scanning and received a monetary compensation. MR data from each subject consist of an isotropic 3D-MPRAGE T1-weighted (0.6 mm^3^) and anisotropic 2D T2-weighted TSE images (0.4 × 0.4 × 2 mm^3^). Images underwent automated correction for intensity non-uniformity, intensity standardization and were linearly registered to the MNI152 space. T1w and T2w images were resampled to a resolution of 0.4 mm^3^. To reduce interpolation artifacts, the T2w data was upsampled using a non-local super-resolution method^28^. For more details about the labeling protocol see the original paper^13^.

#### Winterburn dataset

This dataset contains 5 subjects with 0.3 × 0.3 × 0.3 mm^3^ high resolution T1-weighted and T2-weighted images obtained by 2 × interpolation of 0.6 × 0.6 × 0.6 mm^3^ acquisitions and their corresponding manual segmentations. The HR images are publicly available at the CoBrALab website (http://cobralab.ca/atlases). These MR images were taken from 5 healthy volunteers (2 males, 3 females, aged 29–57). The study was conducted in keeping with the Declaration of Helsinki, was approved by the Centre for Addiction and Mental Health Research Ethics Board, and all subjects provided written, informed consent for data acquisition and sharing. High-resolution T1-weighted images were acquired using the 3D inversion-prepared fast spoiled gradient-recalled echo acquisition (TE/TR = 4.3 ms/9.2 ms, TI = 650 ms, α = 8°, 2-NEX and isotropic resolution of 0.6 mm^3^). High-resolution T2-weighted images were acquired using the 3D fast spin echo acquisition, FSE-CUBE (TE/TR = 95.3 ms/2500 ms, ETL = 100 ms, 2NEX, and isotropic resolution of 0.6 mm^3^). Reconstruction filters, ZIPX2 and ZIP512, were also used resulting in a final isotropic 0.3 mm^3^ dimension voxels. The hippocampus and each of their subfields were segmented manually by an expert rater including 5 labels (CA1, CA2/3, CA4/DG, (SR/SL/SM), and subiculum). For more details about the labeling protocol see the original paper^12^. All methods were performed in accordance to relevant guidelines and regulations.

Example images of these two protocols are shown at Fig. 1. Images and labels were visualized using ITK-SNAP v 3.4.0 software (http://www.itksnap.org).

### Image preprocessing

The images were preprocessed using the following steps: (1) Denoising using the Spatially Adaptive Non-Local Means Filter^29^, (2) Intensity inhomogeneity correction using the N4 bias field correction^30^, (3) Affine registration to the Montreal Neurological Institute (MNI) space by applying the Advanced Normalization Tools (ANTs) package^31^. This registration was estimated using the T1w MNI152 template (at 0.5 mm^3^ resolution) and the T1w images, and applied to both T1w and T2w images (a rigid transformation from T2w to T1w was previously estimated and later concatenated with T1w transformation to perform a single interpolation step when registering both T1w and T2w images). (4) Cropping: To reduce the memory requirements and the computational cost, the images were cropped around HC area, (5) Finally, the cropped images were intensity normalized by subtracting the image mean and dividing by its standard deviation.

### Proposed method

Our proposed method is based on a variant of the well-known UNET architecture^26^. The proposed UNET has 4 resolution levels (from 0.5 to 4 mm). We used three blocks of BatchNormalization, 3D convolution (kernel size of 3 × 3 × 3 voxels) plus ReLU layers for each resolution level. We also used dropout layers (with 0.5 rate) in the encoding part of the UNET to minimize overfitting problems. The input of the network consists of a tensor with two channels (T1 and T2 images). The first resolution level has 64 filters and the next levels multiply by 2 this number to compensate the loss of spatial resolution. Similarly, the number of filters is reduced by 2 in the ascending path of the encoder at each resolution level. The output is also a tensor of *nc* channels represent the probabilities of each subfield and the background.

**Figure 2:**
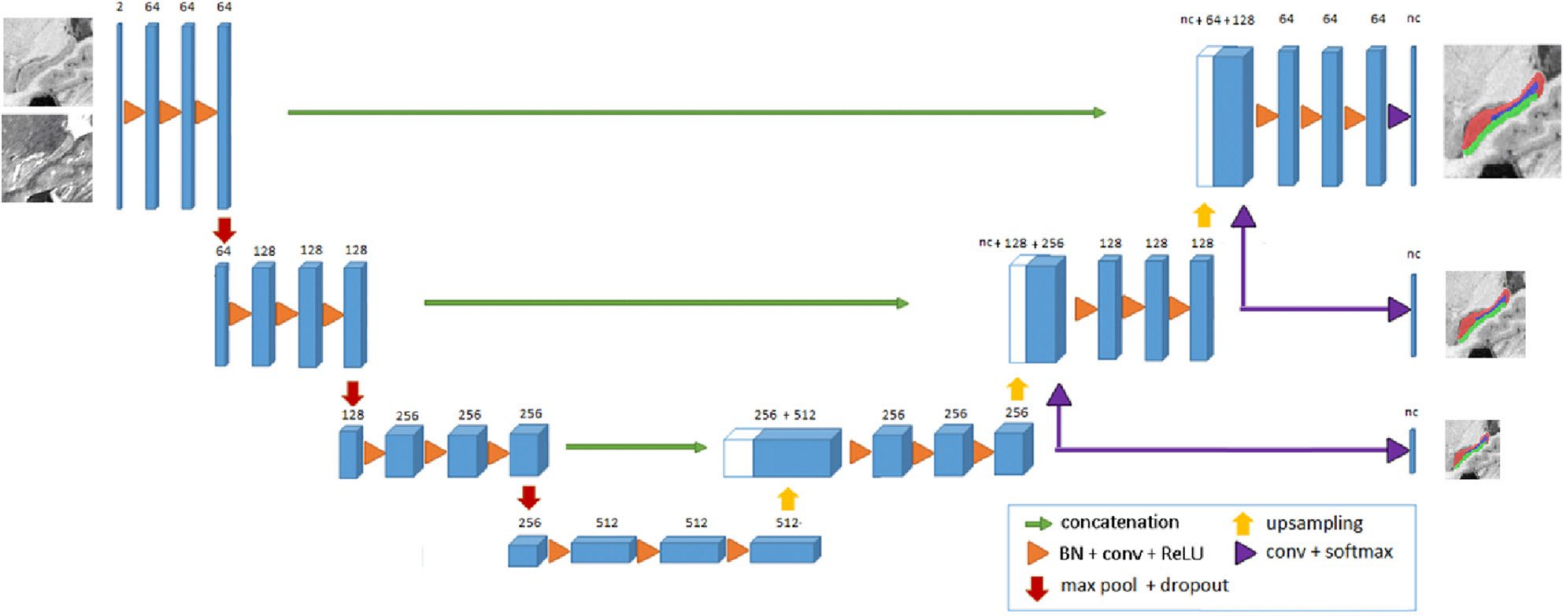
Scheme of the proposed deep supervised UNET CNN.

We also used a modified version of deep supervision^32^ approach that helps to train very deep networks by producing segmentations at different resolution levels. Deep supervision has been shown to not only counteract the adverse effects of gradient vanishing but also to speed up convergence and produce highly accurate results even with limited data. The main difference of our implementation compared to Dou et al., is that we used upsampled low-resolution outputs also as inputs of the next level of the decoder (concatenated with the upsampled features and the encoder shortcut) to help in the next resolution level (only for 1 and 2 mm resolution levels). The resulting network has 56 layers and 35,085,580 trainable parameters. In Fig. 2 the scheme of the proposed network is shown. We will refer to the deep supervised variant of the UNET as DS-UNET3D.

The loss function plays a major role in the training process and an exhaustive search of the most suitable function for the proposed architecture and problem has to be done. One of the most common loss functions for classification is the categorical cross entropy. However, for segmentation purposes it is common to use the dice loss (DL)^33^ as it directly optimizes the segmentation metric most commonly used and it is more robust to the class imbalance problem (1). Recently, a Generalized Dice Loss (GDL)^34^ was proposed to deal with the well-known dependency of the dice index with the size of the labels (2). Inspired by GDL, we propose in this paper the Generalized Jaccard Loss (GDL) (4) which is a variant of Jaccard loss (3) following the same idea to reduce label size dependency:

1 $$DL\left(p,t\right)\underset{}{=1-\frac{2}{\mathrm{NC}}\sum_{\mathrm{c}=1}^{\mathrm{NC}}\frac{\sum_{\mathrm{i}=1}^{\mathrm{N}}{\mathrm{p}}_{\mathrm{ci}}{\mathrm{t}}_{\mathrm{ci}}}{\sum_{\mathrm{i}=1}^{\mathrm{N}}{\mathrm{p}}_{\mathrm{ci}}+{\mathrm{t}}_{\mathrm{ci}}}},$$

2 $$GDL\left(p,t\right)\underset{}{=1-2\frac{\sum_{\mathrm{c}=1}^{\mathrm{NC}}{\mathrm{w}}_{\mathrm{c}}\sum_{\mathrm{i}=1}^{\mathrm{N}}{\mathrm{p}}_{\mathrm{ci}}{\mathrm{t}}_{\mathrm{ci}}}{\sum_{\mathrm{c}=1}^{\mathrm{NC}}{\mathrm{w}}_{\mathrm{c}}\sum_{\mathrm{i}=1}^{\mathrm{N}}{\mathrm{p}}_{\mathrm{ci}}+{\mathrm{t}}_{\mathrm{ci}}}},$$

3 $$JL\left(p,t\right)\underset{}{=1-\frac{1}{\mathrm{NC}}\frac{\sum_{\mathrm{c}=1}^{\mathrm{NC}}\sum_{\mathrm{i}=1}^{\mathrm{N}}{\mathrm{p}}_{\mathrm{ci}}{\mathrm{t}}_{\mathrm{ci}}}{\sum_{\mathrm{c}=1}^{\mathrm{NC}}(\sum_{\mathrm{i}=1}^{\mathrm{N}}{\mathrm{p}}_{\mathrm{ci}}+{\mathrm{t}}_{\mathrm{ci}}-\sum_{\mathrm{i}=1}^{\mathrm{N}}{\mathrm{p}}_{\mathrm{ci}}{\mathrm{t}}_{\mathrm{ci}})}},$$

4 $$GJL\left(p,t\right)\underset{}{=1-\frac{\sum_{\mathrm{c}=1}^{\mathrm{NC}}{\mathrm{w}}_{\mathrm{c}}\sum_{\mathrm{i}=1}^{\mathrm{N}}{\mathrm{p}}_{\mathrm{ci}}{\mathrm{t}}_{\mathrm{ci}}}{\sum_{\mathrm{c}=1}^{\mathrm{NC}}{\mathrm{w}}_{\mathrm{c}}(\sum_{\mathrm{i}=1}^{\mathrm{N}}{\mathrm{p}}_{\mathrm{ci}}+{\mathrm{t}}_{\mathrm{ci}-}\sum_{\mathrm{i}=1}^{\mathrm{N}}{\mathrm{p}}_{\mathrm{ci}}{\mathrm{t}}_{\mathrm{ci}})}},$$

where *N* is the number of voxels, *NC* is the number of classes, *p* is the predicted probability and *t* is the true probability and ${w}_{c}=1/{(\sum_{\mathrm{i}=1}^{\mathrm{N}}{\mathrm{t}}_{\mathrm{ci}})}^{2}$. Alternatively, in our proposed GJL loss we did not use the squared volume to normalize but just the volume, i.e. ${w}_{c}=1/\sum_{\mathrm{i}=1}^{\mathrm{N}}{{\mathrm{t}}_{\mathrm{ci}}}$.

As the size of our training datasets is small (specially for the Winterburn dataset) we used different approaches of data augmentation. We randomly smooth and sharpen the images to simulate different image quality conditions during the training. We expanded the Winterburn dataset with automatic segmentations of the Kulaga-Yoskovitz dataset using the method HIPS. Note that to generate these segmentations only training data was used as library. Finally, we used also mixup^35^ as a data agnostic method for performing data augmentation.

Batch normalization is a highly effective manner to speed up the training process and to improve the results by minimizing the internal covariate shift. However, we realized that when used with small batch sizes it behaves sub-optimally at test time. The reason of this issue is that Batch Normalization layer behaves differently at training and test time. During training, the mean and standard deviation of the activation maps are computed for the whole batch using a moving average estimation to enforce stability during training. However, at test time the network does not processes any batch of data and therefore cannot estimate the mean and standard deviation of the batch, as a result, the network uses the historical mean and standard deviation stored during training. Unfortunately, when using small batch sizes (N = 1 in our case) the stored values do not work very well. Nevertheless, if we run the network in training mode we force the network to use the current mean and standard deviation of the new case and the results are significantly improved. We call this, training time batch normalization (TTBN).

## Experiments and results

In this section, the analysis of the different options of the proposed method and their results are presented. To evaluate the segmentation accuracy, we have used the DICE coefficient^36^ measured in the linear MNI152 space. All experiments were performed using tensorflow 1.2.0 and keras 2.2.4 using Titan Xp Nvidia GPU with 12 GB RAM. To train the network, we used an Adam optimizer^37^ with default parameters during 200 epochs and we test different loss functions with multiscale loss weights (0.1, 0.2 and 0.7) for low, medium and high-resolution outputs respectively (see Fig. 2). A batch size of one was used in all our experiments.

Kulaga-Yoskovitz and Winterburn datasets were preprocessed as described in “Image preprocessing”. To increase the size of the training data, we put together left and right crops by left–right flipping the left crops to generate right oriented crops. This yield 50 right crops in Kulaga-Yoskovitz dataset and 10 right crops in Winterburn dataset. Since the size of both datasets is quite small we have used a K-fold cross validation strategy to increase the relevance of our findings. Specifically, we used K = 5 in both datasets. In Kulaga-Yoskovitz dataset this let each fold with 40 training images (5 of them for validation) and 10 test images. In Winterburn dataset each fold had 8 training images (2 of them for validation) and 2 test images.

### Analysis of the proposed method

There are many factors that affect the performance of deep learning methods such as the architecture, the loss function, data augmentation strategies, etc. In this section we will present some experiments that show their effects on the proposed method.

The first option we tested was the loss function. We compared 5 different loss functions using the same exact network initialization. In Table 1 the validation DICE of each loss function is compared for both datasets. We also included the categorical cross entropy (CCE) in the comparison as it is a common loss used in segmentation/classification. As can be noted, CCE performed worse than dice loss which is a common loss function used in segmentation. Curiously, the GDL failed giving a really low dice compared with the other losses. JL performed similar than dice loss. The proposed GJL was the best performing loss in both datasets and therefore was selected a loss function of the proposed method.

**Table 1:** Average DICE in Kulaga-Yoskovitz (first row) and Winterburn (second row) datasets.

Protocol	CCE	DL	GDL	JL	GJL
Kulaga-Yoskovitz	0.8928 ± 0.0149	0.8952 ± 0.0147	0.7802 ± 0.1445	0.8947 ± 0.0153	**0.8970 ± 0.0143**
Winterburn	0.7124 ± 0.0293	0.7148 ± 0.0285	0.6471 ± 0.0737	0.7135 ± 0.0315	**0.7177 ± 0.0263**

Best results are in bold.

To study the impact of the proposed architecture, we run the proposed network with deep supervision and compared it with the classic UNET. In Table 2 the results of the comparison are shown. As can be seen, the proposed architecture was able to improve the results in both datasets.

**Table 2:** Comparison of our proposed deep supervised UNET vs classic UNET 3D.

Protocol	UNET 3D	DS-UNET 3D
Kulaga-Yoskovitz	0.8970 ± 0.0143	**0.9001 ± 0.0130**
Winterburn	0.7177 ± 0.0263	**0.7202 ± 0.0288**

﻿Best results are in bold.

It is well-known that in deep learning the amount of training data plays a major role (probably the biggest) in the quality of the network results. Unfortunately, manually labeled cases of hippocampal subfields is a rare resource due to the difficulty of generating such data. Automatic data augmentation has been traditional used to artificially increase the number of training cases. This has been usually done applying random transformations on the available training data (rotation, scale, etc.). In this project, we have used a combination of different methods to augment the number of training cases. In the case of Kulaga-Yuskevitz, we randomly smooth and sharpen the cropped images to generate low and high-quality images to improve generalization capabilities of the network. We also used mixup^35^ to linearly combine inputs and outputs (alfa = 0.3). Mixup is a data-agnostic data augmentation method that has been proven beneficial specially when using a small training dataset^38^. In the case of Winterburn dataset, we used the same approach but in addition we increased the training dataset using automatic segmentations of the Kulaga-Yuskevitz dataset with the HIPS method^18^ which is a patch-based multi-atlas label fusion based method (using as atlases the training cases of each fold). In the Table 3 the results of the proposed method with and without data augmentations are shown. As expected, data augmentation strategies helped to improve the results in both datasets. The improvement in Winterburn dataset was more important given the small size of the training set (N = 6).

**Table 3:** Data augmentation results.

Protocol	No data augmentation	Data augmentation
Kulaga-Yoskovitz	0.9001 ± 0.0130	**0.9025 ± 0.0130**
Winterburn	*0.7202* ± *0.0288*	**0.7354 ± 0.0240**

Best results are in bold.

A last experiment was performed to evaluate the effect of the TTBN technique. In Table 4 the results of both datasets are shown. As can be seen, TTBN helped in both data sets, but the improvement in the Winterburn dataset was relatively greater.

**Table 4:** Training time batch normalization results.

Protocol	Standard prediction	TTBN prediction
Kulaga-Yoskovitz	*0.9025* ± *0.0130*	**0.9037 ± 0.0129**
Winterburn	*0.7354* ± *0.0240*	**0.7418 ± 0.0188**

Best results are in bold.

**Figure 3:**
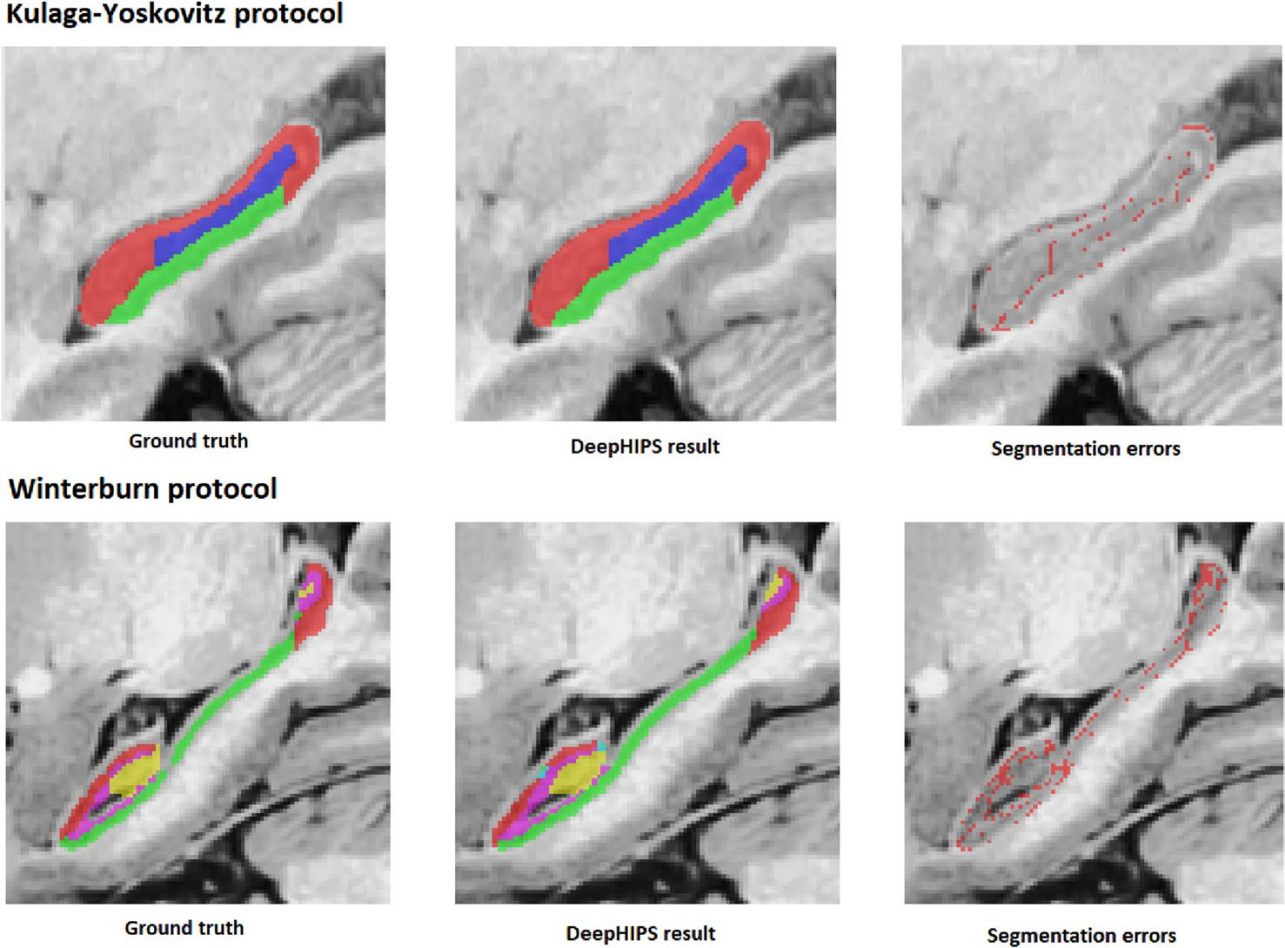
Example results of Winterburn and Kulaga-Yoskovitz protocol automatic segmentation using DeepHIPS. Images generated using ITK-SNAP v 3.4.0.

For the final results of both datasets, we estimated each structure dice, average dice among structures and whole hippocampus dice. In Table 5 the k-fold cross validation results for both datasets are shown. An example result of DeepHIPS for both protocols is shown in Fig. 3.

**Table 5:** Mean DICE and standard deviation for each structure segmentation over the Kulaga-Yoskovitz and Winterburn datasets.

Structure\protocol	Kulaga-Yoskovitz	Structure\protocol	Winterburn
Average	0.9037 ± 0.0129	Average	0.7418 ± 0.0188
CA1-3	0.9245 ± 0.0106	CA1	0.7805 ± 0.0170
CA4\DG	0.8887 ± 0.0237	CA2\CA3	0.6686 ± 0.0436
Subiculum	0.8980 ± 0.0155	CA4\DG	0.8096 ± 0.0301
		SR\SL\SM	0.7066 ± 0.0197
		Subiculum	0.7439 ± 0.0338
Hippocampus	0.9618 ± 0.0051	Hippocampus	0.9123 ± 0.0106

### Standard resolution vs high resolution

The proposed method uses high resolution MR images but these sequences are not always available either in research or in clinical environments. However, it would be desirable to be able to analyze legacy data. For this reason, we evaluated the proposed method using standard resolution (1 × 1 × 1 mm^3^) images upsampled to 0.5 × 0.5 × 0.5 mm^3^ using B-spline interpolation and a super-resolution technique^28^. To do it, we reduced the resolution of the HR images by a factor 2 and later we upsampled them using the described methods.

Tables 6 and 7 show the results for both datasets. The results confirm that the proposed method can produce competitive results when using standard resolution images. Note that the results using LASR are better than using B-spline interpolation for both datasets and closely resembles those obtained using the original HR images. This important result shows that the proposed framework can efficiently process usual 1 × 1 × 1 mm^3^ MR data. Recent advances in deep learning based superresolution^39^ can further reduce the gap between original HR data results and the upsampled standard resolution images. However, this is beyond the objectives of the work and will be studied in a future research.

**Table 6:** Winterburn dataset mean DICE and standard deviation for each structure segmentation using the B-spline interpolation and LASR to the previously downsampled image to be segmented. Best results in bold. Results using the HR images are also provided for comparison.

Structure	B-spline	LASR	HR
Average	0.7218 ± 0.0239	0.7317 ± 0.0216	**0.7418 ± 0.0188**
CA1	0.7252 ± 0.0183	0.7738 ± 0.0167	**0.7805 ± 0.0170**
CA2\CA3	0.6376 ± 0.0606	0.6504 ± 0.0529	**0.6686 ± 0.0436**
CA4\DG	0.7943 ± 0.0333	0.8001 ± 0.0314	**0.8096 ± 0.0301**
SR\SL\SM	0.6837 ± 0.0178	0.6916 ± 0.0199	**0.7066 ± 0.0197**
Subiculum	0.7251 ± 0.0411	0.7424 ± 0.0344	**0.7439 ± 0.0338**
Hippocampus	0.9049 ± 0.0097	0.9095 ± 0.0100	**0.9123 ± 0.0106**

**Table 7:** Kulaga-Yoskovitz dataset mean DICE and standard deviation for each structure segmentation using the B-spline interpolation and LASR to the previously downsampled image to be segmented. Best results in bold. Results using the HR images are also provided for comparison.

Structure	BSpline		LASR		HR	
Average	0.8957 ± 0.0135		0.8988 ± 0.0134		**0.9037 ± 0.0129**	
CA1-3	0.9171 ± 0.0107		0.9203 ± 0.0102		**0.9245 ± 0.0106**	
CA4/DG	0.8787 ± 0.0253		0.8823 ± 0.0259		**0.8887 ± 0.0237**	
Subiculum	0.8913 ± 0.0163		0.8937 ± 0.0167		**0.8980 ± 0.0155**	
Hippocampus	0.9571 ± 0.0056		0.9593 ± 0.0065		**0.9618 ± 0.0051**	

### Method comparison

The proposed method was compared with state-of-the-art related methods. Specifically, for the Kulaga-Yoskovitz dataset we compared with HIPS method^18^ and a recent deep learning-based method named ResDUnet dedicated to hippocampus subfields segmentation^27^. In both cases we used published results in their papers for the comparison. In Table 8 we show the results of the comparison. We included also the inter and intra-rater accuracy for comparison purposes. As can be noticed, the proposed method outperformed previous state-of-the-art methods. It is also worth to note that the proposed method improved the inter-rater accuracy and got very close to the intra-rater accuracy.

**Table 8:** Mean DICE and standard deviation for each structure segmentation over the Kulaga-Yoskovitz dataset. Best results in bold.

Structure	HIPS	ResDUnet	Proposed	Inter-rater	Intra-rater
Average	0.8879	0.8960	**0.9037**	0.8833	0.9113
CA1-3	0.9158 ± 0.0150	0.9200 ± 0.0110	**0.9245 ± 0.0106**	0.8760 ± 0.048	0.9290 ± 0.010
CA4\DG	0.8863 ± 0.0340	0.8790 ± 0.0200	**0.8887 ± 0.0237**	0.9030 ± 0.036	0.9000 ± 0.019
Subiculum	0.8616 ± 0.0210	0.8880 ± 0.0160	**0.8980 ± 0.0155**	0.8710 ± 0.053	0.9050 ± 0.016
Hippocampus	0.9595	–	**0.9618**	–	–

*For the Winterburn dataset,* we compared with HIPS method^18^ that represents the state of the art in this dataset. In Table 9, we show the results of the comparison. We included also the intra-rater accuracy for comparison purposes. As can be noticed the proposed method outperformed HIPS method by a large margin and got very close to the intra-rater accuracy.

**Table 9:** Mean DICE in the MNI space and standard deviation for each structure segmentation using high resolution T1w, T2w and Multispectral respectively over the Winterburn dataset. Best results in bold.

Structure	HIPS	Proposed	Intra-rater
Average	0.7158	**0.7418**	0.742
CA1	0.7762 ± 0.0251	**0.7805 ± 0.0170**	0.780
CA2\CA3	0.6179 ± 0.0630	**0.6686 ± 0.0436**	0.640
CA4\DG	0.7750 ± 0.0307	**0.8096 ± 0.0301**	0.830
SR\SL\SM	0.7018 ± 0.0191	**0.7066 ± 0.0197**	0.710
Subiculum	0.7082 ± 0.0597	**0.7439 ± 0.0338**	0.750
Hippocampus	0.9111	**0.9123**	0.910

Regarding to the execution time, the proposed network takes around 1 s to segment a new case. The whole DeepHIPS pipeline (including preprocessing) takes around 2 min while HIPS method takes around 20 min.

## Discussion

In this paper, we have presented a new deep learning-based method for HR hippocampus subfield segmentation that we called DeepHIPS. We have validated the proposed method using 2 publically available datasets (Winterburn and Kulaga-Yoskovitz).

Our proposed method first preprocesses the HR T1 and T2 images to improve their quality and to locate them into a standard space (MNI152) to finally crop the region of interest to process. From the architecture point of view, our model is a 3D UNET variant that uses deep supervision and low-resolution feedback to make easier the training process. We found that this variant worked better that the classic UNET.

We have also proposed a novel loss function (GJL) based on the Jaccard similarity index that enables to improve the accuracy of the network borrowing ideas from a modified version of the GDL (i.e. using linear volume weights instead of quadratic). We further improve the results using classical data augmentation techniques such as image mirroring and intensity transformation and more modern ones such as mixup.

Finally, we improved the results of the network at test time by running Batch normalization layers in training mode instead of test mode. We found that when using small batch sizes (N = 1 in our case) batch normalization layers didn’t behave properly due to the use of the stored mean and standard deviation during training. Using current sample statistics systematically improved the results in all our experiments despite the simplicity of the approach (especially in the Winterburn dataset with an improvement of nearly the 9%). We called this strategy Training Time Bach Normalization (TTBN).

We compared the results of the proposed method with state-of-the-art methods in two datasets. In the Kulaga-Yoskovitz dataset we compared with HIPS method and a recent deep learning-based method named ResDUnet. The proposed method improved the results of both methods for all subfields and got closer to the intra-rater accuracy which can be considered as the upper bound of the method. For the Winterburn dataset, we compared with HIPS method and again, the proposed method improved the results for all subfields and the overall accuracy got very close to the intra-rater accuracy.

We also studied the accuracy of the propose method using standard resolution images (1 mm^2^) upsampled to HR (0.5 mm^2^) using superresolution method (LASR). Although the accuracy slightly dropped compared to the HR results we found it still very competitive making possible the use of legacy data.

We are aware that the training libraries of the proposed method are quite small to ensure a good generalization (especially in the case of Winterburn) and our future efforts will be directed to increase the size of these libraries by manually labeling new cases and using semi-supervised approaches to automatically extend the training dataset size.

From an efficiency point of view the proposed method is not only more accurate but also more efficient than previous state of the art (HIPS) reducing by a factor 10 the total execution time.

## Conclusion

In this work, we have presented a new method for HR hippocampus subfield segmentation based on a deep learning approach and we have validated it with two publically available datasets (Winterburn and Kulaga-Yoskovitz) showing competitive results in both accuracy and efficiency. We plan to make fully accessible the DeepHIPS pipeline through the new release of our online image analysis service volbrain (http://volbrain.upv.es) so researchers around the world can use our pipeline without requiring complex pipeline installations or the use of expensive hardware (GPUs, etc.).
